# Management of Follow-Up With Preterm Infants During the Outbreak in China

**DOI:** 10.3389/fped.2021.637275

**Published:** 2021-04-29

**Authors:** Linlin Li, Zhenghong Li, Weilin Wan, Ji Li, Yu Zhang, Changyan Wang, Lin Wang

**Affiliations:** Department of Pediatrics, State Key Laboratory of Complex Severe and Rare Diseases, Peking Union Medical College Hospital, Chinese Academy of Medical Science and Peking Union Medical College, Beijing, China

**Keywords:** follow-up, preterm infant, COVID-19, online, face-to-face

## Abstract

**Introduction:** Coronavirus disease 2019 (COVID-19) swept Wuhan in January 2020. Other cities in China also suffered during the pandemic. Routine medical services were conducted in the Neonatal Intensive Unit (NICU) as usual, but the follow-up after discharge was seriously affected.

**Objective:** To investigate the feasibility and effectiveness of a combination of online and face-to-face follow-up for preterm infants during the COVID-19 epidemic and to explore a follow-up pattern that can provide follow-up services while maximizing the protection of preterm infants and soothing the fear of their parents.

**Methods:** Preterm infants (*n* = 35) whose first follow-up appointment was scheduled from February 1 to April 30, 2020, and preterm infants (*n* = 43) in the NICU follow-up group who were discharged from January 1, 2018, to January 31, 2020, who had a second or later routine follow-up appointment scheduled from February 1 to April 30, 2020, were enrolled. We provided a combination of online and face-to-face follow-up for preterm infants surveyed with the Wenjuanxing platform before and after the online follow-up and compared the first-time follow-up rate between the outbreak and the same period of the previous year.

**Results:** Feeding and oral medicine and supplements were the most concerning problems of the parents of preterm infants. The anxiety level of the family was significantly decreased after online follow-up (*P* < 0.05). A total of 96.8% of parents were satisfied or very satisfied with online follow-up, and 95.2% of parents thought that online follow-up had answered all their questions. Only 35.5% of parents thought online follow-up could replace face-to-face follow-up.

**Conclusion:** The combination of online and face-to-face follow-up alleviated the anxiety of the parents during the outbreak and achieved a similar first-time follow-up rate as the same period in 2019.

## Introduction

The immaturity of organs and systems and central nervous system sequelae affect the early development, quality of life and long-term prognosis of premature infants (1–5). Survival, therefore, is only the first step: comprehensive improvement of prognosis is the ultimate objective for preterm infants. To ensure early and timely intervention for preterm infants after discharge, the general office of the National Health and Family Planning Commission of China issued the “Norms for the Health Care of Preterm Infants” in 2017 and put forward suggestions and requirements for the frequency and content of preterm infant follow-up after discharge. However, the sudden outbreak of coronavirus disease 2019 (COVID-19) made routine follow-up of preterm infants difficult to continue. COVID-19 pneumonia is an infectious disease. All populations, including children, are susceptible to it (6). The condition added more stress for preterm infants' parents, who were already worried about infection since the immune function of their infants was immature. All these factors hampered follow-up with preterm infants during the epidemic. We conducted a prospective study to investigate the feasibility and effectiveness of a combination of online and face-to-face follow-up and to explore a follow-up method that can protect preterm infants and soothe the anxiety of the parents of preterm infants during the outbreak.

## Methods

### Study of Combination of Online and Face-to-Face Follow-Up

COVID-19 swept Wuhan in January 2020. Other cities in China also suffered during the pandemic. Routine medical care for preterm infants was conducted in the NICU as usual, but follow-up after hospital discharge almost stopped. To guarantee follow-up with preterm infants and relieve parents' anxiety, we combined online follow-up and face-to-face follow-up and conducted a prospective study to investigate the feasibility and effectiveness of the combination of online and face-to-face follow-up.

#### Study Population

Inclusion criteria: (1) gestational age <37 weeks; (2) length of hospital stay ≥3 days; (3) discharged as planned from the NICU of Peking Union Medical College Hospital (PUMCH); (4) preterm infants whose first follow-up appointment was scheduled from February 1 to April 30, 2020, or preterm infants in the NICU follow-up group who were discharged from January 1, 2018, to January 31, 2020, and whose second or later routine follow-up appointment(s) was scheduled from February 1 to April 30, 2020.

Exclusion criterion: infants who died after discharge.

#### Process of Combining Online and Face-to-Face Follow-Up

Before the pandemic, we followed up with preterm infants face-to-face 2 weeks after they were discharged, then every month before a corrected age (CA) of 6 months and every two months between ages 6 and 12 months.

We used WeChat and Wenjuanxing to conduct online follow-up with preterm infants. WeChat is an app that supplies text and voice messaging communication services and supports video chat. WeChat was used to contact parents of preterm infants and implement online follow-up. Wenjuanxing is a professional online questionnaire survey, evaluation and voting platform that was used to administer the survey questionnaire before and after online follow-up.

a. Establish a connection between the infants' parents and a clinic nurse

We let the parents scan the WeChat QR code to make appointments for the first follow-up before the preterm infants were discharged from the NICU. We also informed the parents in our NICU 2018~2020 discharged preterm infants WeChat group that we provided online follow-up service during the epidemic, if needed.

b. Arrange online follow-up

According to the discharge order (which provided suggested follow-up frequency after discharge), diagnosis (including a recommendation for a doctor who was skilled in the relevant field), and previous follow-up doctors, the clinic nurse made online follow-up appointments for the infants after discussing the date and time with the parents via WeChat. The parents could choose to visit the clinic as well. The parents who preferred online service were informed about the follow-up instructions, including date and time, doctor's name, and contact information. Informed consent for the purpose and limitations of online follow-up and video of measuring methods of height, weight and head circumference were sent via WeChat to the parents before the online service.

c. Administer survey questionnaire before online follow-up

The parents were prompted to scan the QR code of the pre–follow-up questionnaire in Wenjuanxing and complete it anonymously. The questionnaire was self-completed by mothers of infants outside the hospital to ensure anonymity and confidentiality. The contents of the questionnaire included (1) basic information of preterm infants (sex, birth weight, gestational age and date of birth); (2) concerns of the parents (feeding, oral medicine and supplements, laboratory examination, development, early education, growth, excretion, jaundice, hernias, skin care and respiration) and the degree of concern (not concerned at all, not concerned, a little concerned, concerned, strongly concerned); and (3) the anxiety level of the mothers, other family members, and the whole family (not anxious at all, not anxious, slightly anxious, anxious, very anxious).

d. Send parent reminders

To prevent loss to follow-up or missed appointments, the clinic nurse reminded the parents via WeChat 1 day before the follow-up date.

e. Conduct online follow-up

We conducted online follow-up via telephone and WeChat. Online follow-up was conducted by the same group of doctors who conducted face-to-face follow-up with preterm infants before the epidemic. Doctors evaluated the babies' development status, feeding, excretion, nutrient supplementation and vaccination through telemedicine visits and then provided corresponding guidance. Neurodevelopmental assessment and early development promotion guidance were implemented through WeChat video. We addressed preterm infant pathologies through follow-up after discharge from the NICU. Both online and face-to-face follow-up included growth monitoring and evaluation, feeding consultation and guidance, nutrition supplementation, nursing and disease prevention guidance, and early development promotion guidance. For physical examinations, neuropsychological and behavioral development monitoring and evaluation, which cannot be completed online, doctors asked parents to describe the observation of the baby through daily life and demonstrate whether the baby could do some milestone activities via the WeChat video call while the online follow-up was conducted. For example, doctors could observe the muscle tone when the parents were asked to move the baby's legs and observe whether the baby could raise his/her head or turn over via a WeChat video call. Parents were informed to take their infants to see doctors face-to-face for diagnosis and treatment of ailments if the baby was suspected to have high muscle tone or delayed neuropsychological and behavioral development.

f. Administer survey questionnaire after online follow-up

Families were asked to complete a survey questionnaire about the anxiety of the family and their attitude toward online follow-up after the service. Parents of the infants completed the survey questionnaire anonymously; the questionnaires were self-completed by mothers of infants outside the hospital to ensure anonymity and confidentiality. The contents of the questionnaire included (1) basic information (sex, birth weight, gestational age and the date of birth); (2) anxiety level of mothers, other family members, and the whole family (not anxious at all, not anxious, slightly anxious, anxious, very anxious); (3) attitude toward online follow-up: whether all the parents' questions had been answered and if online follow-up could replace face-to-face follow-up (strongly disagree, disagree, neutral, agree, strongly agree); and (4) satisfaction with online follow-up (strongly dissatisfied, dissatisfied, neutral, satisfied, strongly satisfied).

#### Data Collection

We collected basic information on the enrolled preterm infants, including gestational age, the date of birth and admission, the expected date of birth, birth weight, sex, the date of discharge, and the length of hospital stay, using the electric medical record system. Parents of the preterm infants entered the infants' name, sex, ID, gestational age, corrected age, height, weight and head circumference into Wenjuanxing before online follow-up. Parents assessed their issues of concern prior to follow-up, including feeding, oral medicine and supplements, laboratory examination, development, early education, growth, excretion, jaundice, hernias, skin care and respiration using a 5-point Likert scale. We also used a 5-point Likert scale to assess the satisfaction of the parents with online follow-up and their level of agreement with the statements “online follow-up answered all my questions” and “online follow-up could replace face-to-face follow-up” after online follow-up. We also asked them to rate the anxiety level of the mother, other family members and the whole family of preterm infants before and after online follow-up via Wenjuanxing.

### Comparison of the Combination of Online and Face-to-Face Follow-Up With Face-to-Face Follow-Up Only

To evaluate the effectiveness of the first-time follow-up using the combination of online and face-to-face follow-up during the outbreak, we compared the first-time follow-up rate of the preterm infants seen between February 1 to April 30, 2020, and February 1 to April 30, 2019.

#### Study Population

Inclusion criteria: (1) gestational age <37 weeks; (2) a length of hospital stay ≥3 days; and (3) discharge as planned from the NICU of PUMCH.

Exclusion criterion: infants who died after discharge.

**2020 group**: Preterm infants discharged from the NICU between February 1 and April 30, 2020. We provided online follow-up service during this special period. However, we also made appointments for those who had to visit the clinic for blood tests or imaging examinations.

**2019 group**: Preterm infants discharged from the NICU between February 1 and April 30, 2019. All received face-to-face follow-up.

#### Data Collection

We prospectively collected the data of the 2020 group, including gestational age, the date of birth and admission, the expected date of birth, birth weight, sex, the date of discharge, the length of hospital stay and the date of the first face-to-face or online follow-up appointment. We retrospectively collected the same data from the first follow-up for the 2019 group through the medical record system.

### Statistical Analysis

All the data in Wenjuanxing were converted into SPSS or Excel. Data were analyzed with SPSS Version 25.0 (SPSS, Inc., Chicago, Illinois, USA). Categorical data are presented as frequency distributions and proportions; comparisons between groups were made using the chi square test. Continuous data are presented as the mean ± SD or median (p25 ~ p75), and comparisons between the two groups were made using two independent sample t-tests or rank sum tests.

## Results

### Study of Combination of Online and Face-to-Face Follow-Up

#### Demographics

There were 37 preterm infants whose first-time follow-up was scheduled between February 1 and April 30, 2020. In addition, 35 (94.6%) patients received follow-up within 3 weeks after discharge: 21 patients received follow-up online, and 14 patients received face-to-face follow-up. The gestational age of the infants who received follow-up online was 33.17 ± 3.08 w, the birth weight was 1993.57 ± 745.30 g, and the length of hospital stay was 10 (5.5, 36.5) days. For those who received face-to-face follow-up, the gestational age was 33.91 ± 2.72 w, the birth weight was 2148.57 ± 67.50 g, and the length of hospital stay was 12 (6.75, 32.75) days.

We also informed 45 parents in the NICU 2018~2020 discharged preterm infants WeChat group for second or later routine follow-up. Among them, 41 patients received follow-up online, two patients visited the clinic, and two patients were lost to follow-up. The gestational age of those infants who received follow-up online was 32.12 ± 3.21 w, the birth weight was 1766.71 ± 745.55 g, and the corrected age was 4 (1.5, 9) months.

**Figure 1:**
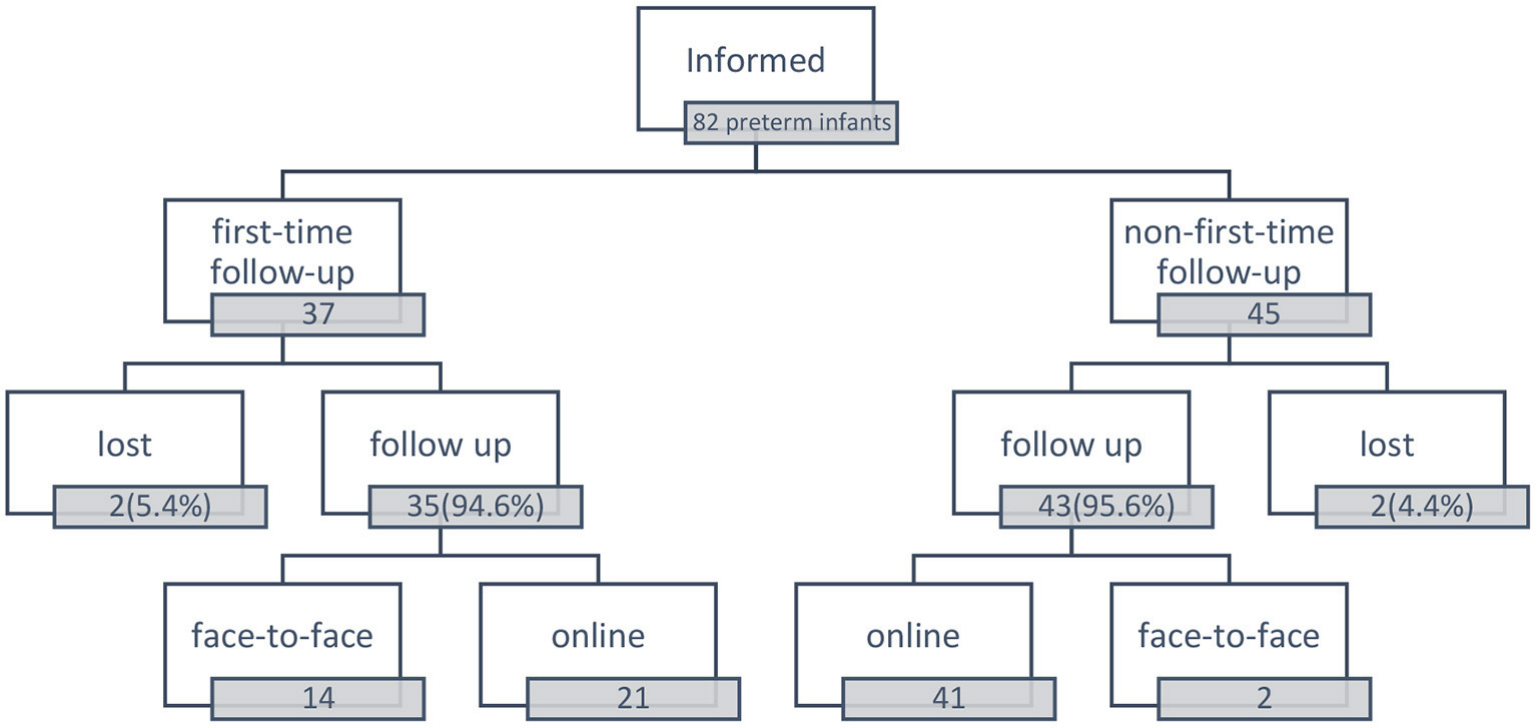
Follow-up situation of preterm infants.

Overall, 62 patients were enrolled in online follow-up (Figure 1).

### Main Concerns of the Parents of Preterm Infants Before Online Follow-Up

**Figure 2:**
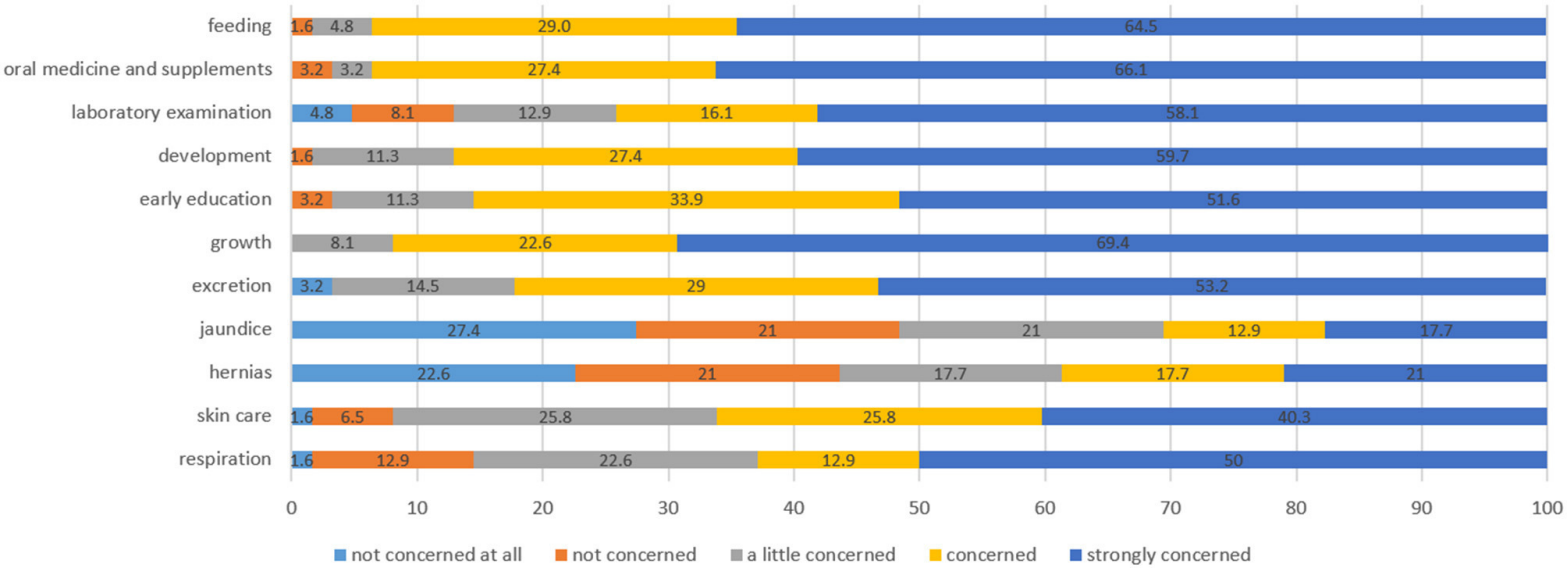
Main concerns of the parents of preterm infants (%).

Before online follow-up, we sent out 62 Wenjuanxing questionnaires; all were completed and returned. Feeding and oral medicine and supplements were the issues that parents were most concerned about. A total of 93.5% of parents were strongly concerned or concerned about these two issues. Growth is another problem parents worried about; none indicated that they were not concerned about it at all (Figure 2).

#### Anxiety Level of Mothers and Families of Premature Infants

We issued 62 questionnaires about the anxiety of mothers and families via Wenjuanxing before and after online follow-up and retrieved all of them. We observed that the anxiety levels of the mothers, other family members and the whole family significantly declined after online follow-up (*P* < 0.05) (Table 1).

**Table 1:** Anxiety level of the mothers and families of preterm infants.

**Anxiety**	**Before follow-up**	**After follow-up**	***Z***	***p*-value**
	***N* (%)**	***N* (%)**		
Anxiety level of mothers			−4.857	0
Not anxious at all	6(9.7%)	16 (25.8%)		
Not anxious	16(25.8%)	27 (43.5%)		
Slightly anxious	27(43.5%)	15 (24.2%)		
Anxious	7(11.3%)	4 (6.5%)		
Very anxious	6(9.7%)	0 (0%)		
Anxiety level of other family members			−5.169	0
Not anxious at all	7(17%)	17 (27.4%)		
Not anxious	17(27,4%)	31 (50.0%)		
Slightly anxious	27(43.5%)	11(17.7%)		
Anxious	7(11.3%)	3 (4.8%)		
Very anxious	4(6.5%)	0 (0%)		
Anxiety level of the whole family			−4.796	0
Not anxious at all	7(17%)	17(27.4%)		
Not anxious	17(27,4%)	29 (46.8%)		
Slightly anxious	28(45.5%)	13 (21.0%)		
Anxious	8 (12.9%)	3 (4.8%)		
Very anxious	2 (3.2%)	0 (0%)		

#### Parent Satisfaction With Online Follow-Up and Levels of Agreement With the Statements “Online Follow-Up Answered All My Questions” and “Online Follow-Up Could Replace Face-to-Face Follow-Up”

**Figure 3:**
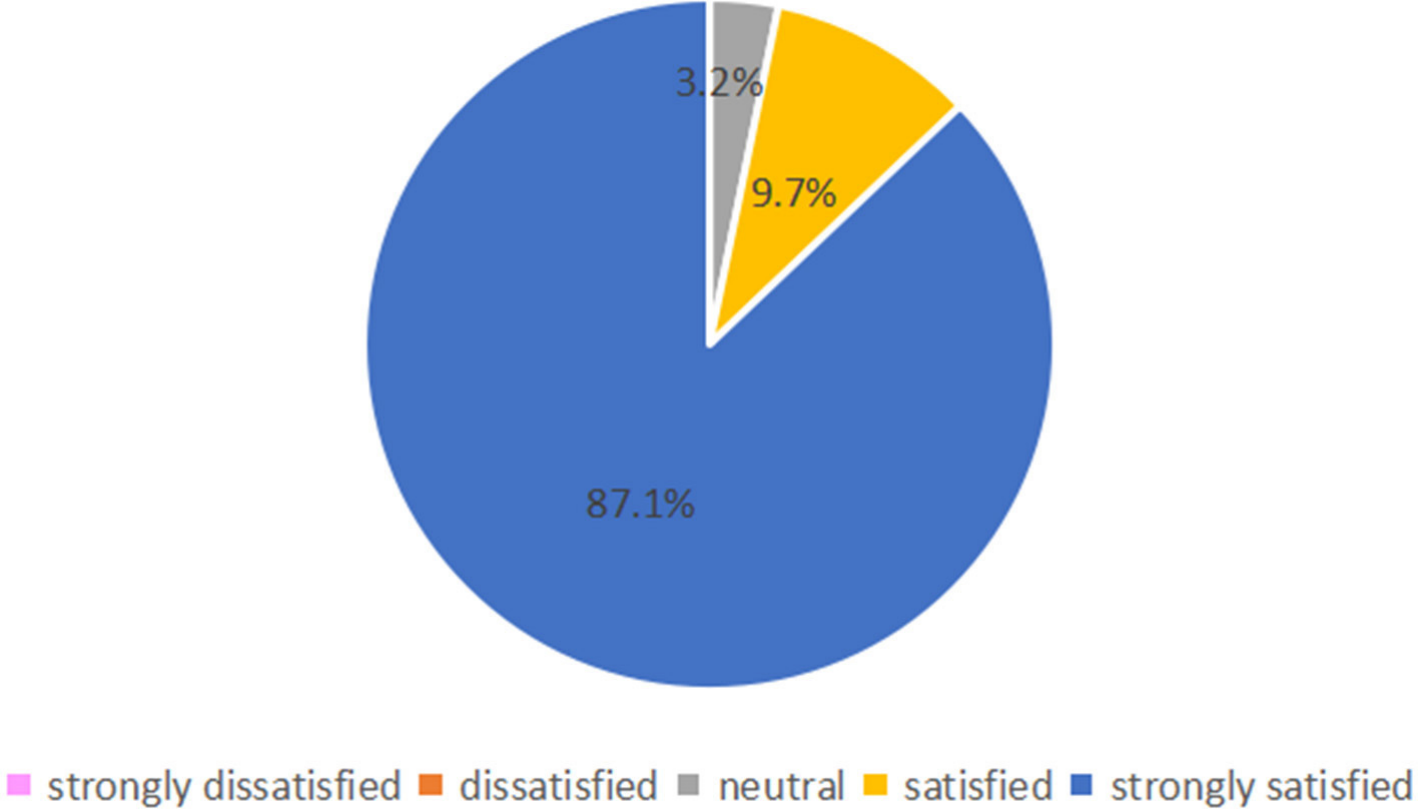
Satisfaction of parents of the preterm infants with online follow-up.

**Figure 4:**
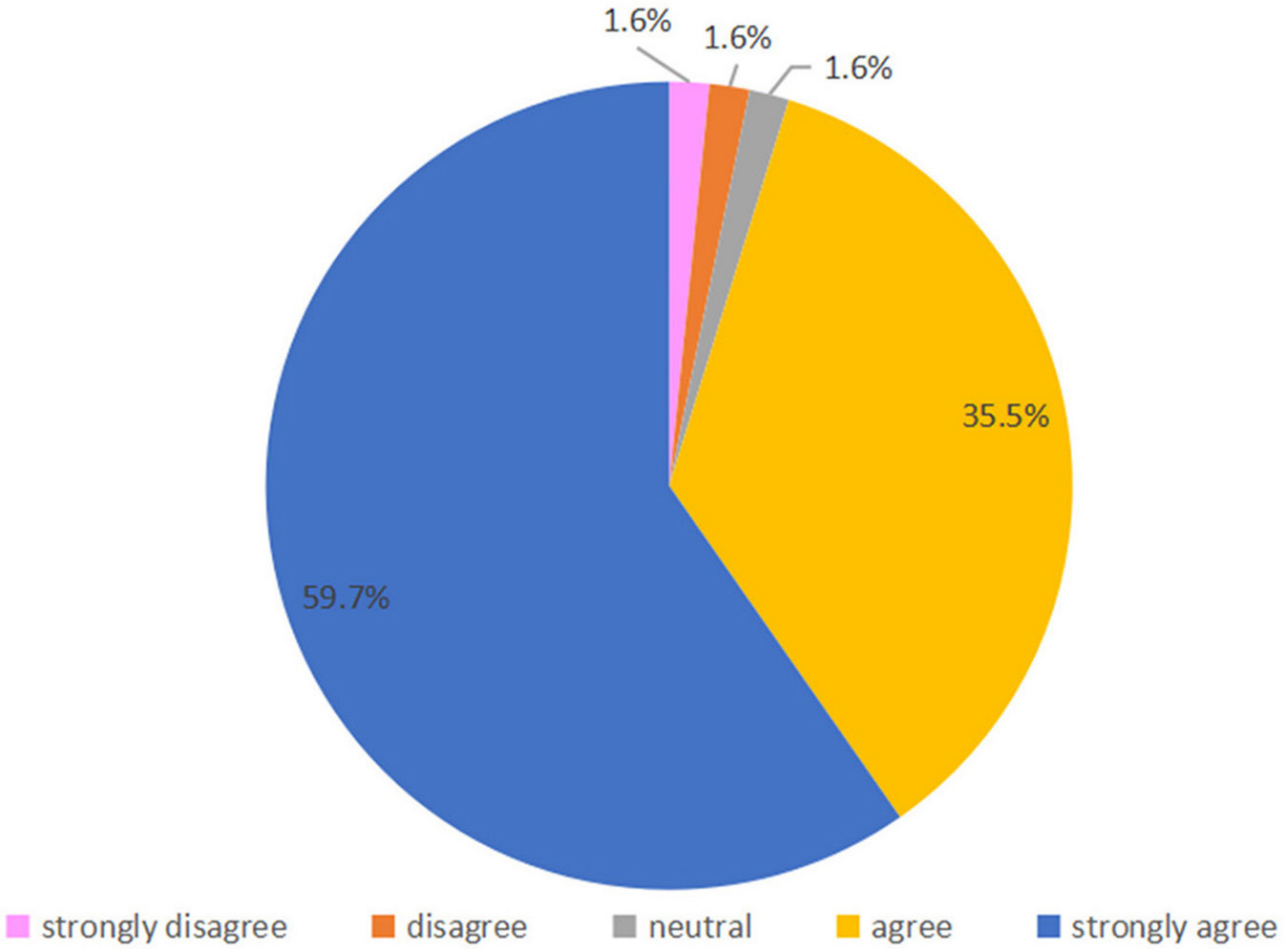
Attitude toward “Online follow-up answered all my questions”.

**Figure 5:**
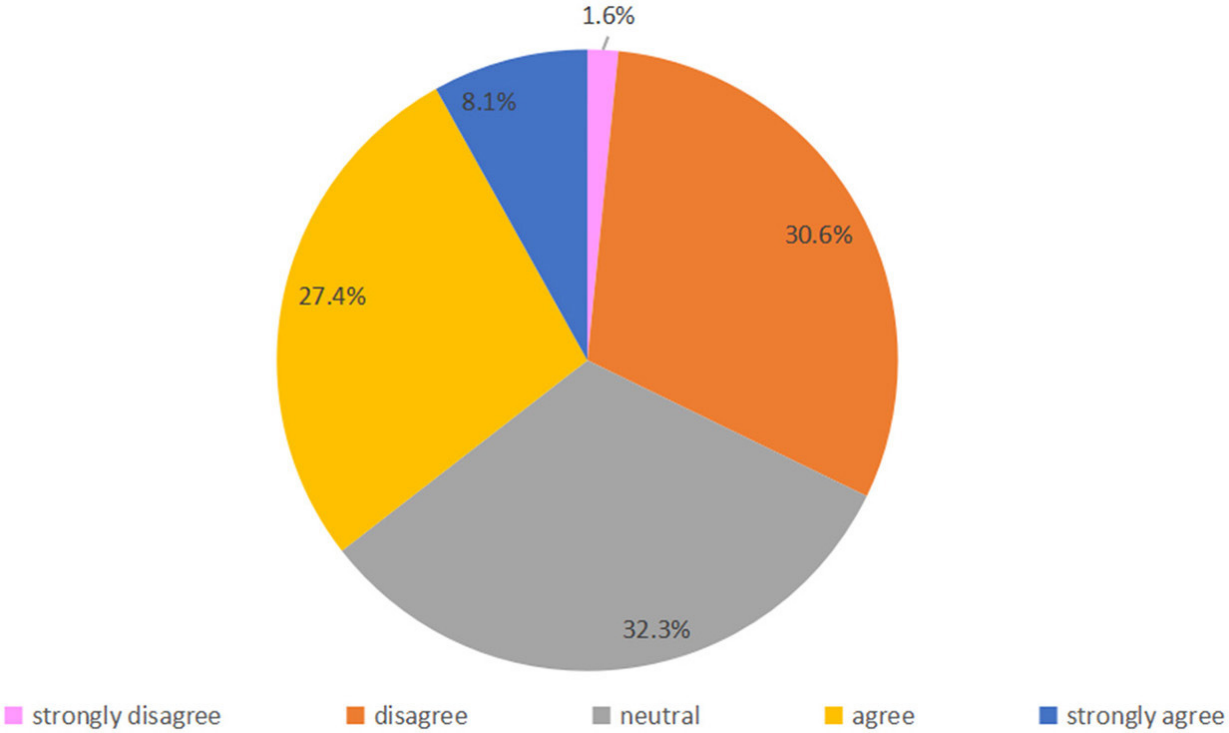
Attitude toward “Online follow-up could replace face-to-face follow-up”.

Fifty-four parents (87.1%) were strongly satisfied with online follow-up, and 6 (9.7%) were satisfied; only 2 (3.2%) were satisfied neutral, and none were dissatisfied or strongly dissatisfied (Figure 3). Regarding their agreement with the statement “online follow-up answered all my questions,” 22 (35.5%) and 37 (59.7%) parents selected “agree” or “strongly agree,” respectively, and 1 (1.6%) parent voted strongly disagree, disagree and neutral respectively (Figure 4). Regarding agreement with “the online follow-up could replace face-to-face follow-up,” 1 (1.6%) parent strongly disagreed, 19 (30.6%) disagreed, 20 (32.3%) were neutral 17 (27.4%) agreed and 5 (8.1%) strongly agreed (Figure 5).

### Comparison of Combination Online and Face-to-Face Follow-Up and Face-to-Face Follow-Up Only

#### Demographics

A total of 42 patients in the 2019 group and 37 patients in the 2020 group were enrolled. There were no significant differences in characteristics between the two groups (*p* > 0.05) (Table 2).

**Table 2:** Characteristics of preterm infants.

	**2019**	**2020**	**Statistics**	***p*-value**
	***n* = 42**	***n* = 37**		
Sex			0.908^a^	0.341
Male	24 (57.1%)	25 (67.6%)		
Female	18 (42.9%)	12 (32.4%)		
Birth weight(g)	2070.71 ± 581.42	2114.59 ± 683.33	−0.308^b^	0.759
Gestational age (w)	34.57 (33.67, 35.76)	35 (32.5, 35.64)	−0.221^c^	0.825
Length of stay (d)	7 (5, 17.5)	10 (5.5,30)	−1.144	0.253
Home address			1.613^a^	0.204
In Beijing	33 (78.6%)	33 (89.2%)		
Out of Beijing	9 (21.4%)	4 (10.8%)		

*a, χ^2^; b, t; c, z*.

#### Follow-Up Rate of Preterm Infants After Discharge

Among the 35 preterm infants who received follow-up within 3 weeks after discharge in the 2020 group, 21 received follow-up online, and 14 received face-to-face follow-up. There were no significant differences in the follow-up rate of preterm infants within 3 weeks after discharge between the two groups (*p* > 0.05) (Table 3).

**Table 3:** Follow-up rate of the preterm infants within 3 weeks after discharge.

**Group**	**Cases**	**Follow up within 3 weeks**	
		**Yes**	**No**
2019	42	37 (88.1%)	5 (11.9%)
2020	37	35 (94.6%)	2 (5.4%)
Continuity correction χ^2^		0.382	
*p*-value		0.537	

## Discussion

Scheduled follow-up after discharge can improve the prognosis of preterm infants (7, 8). In contrast, the incidence of motor and nervous system problems and cognitive skills of preterm infants who did not receive follow-up were higher than those who received follow-up on time (9). At the peak of the epidemic in China, face-to-face follow-up of preterm infants was disrupted because of the closure of some clinical services. In our study, the survey before online follow-up showed that 35.5% of mothers of preterm infants were anxious or very anxious because they worried that their infants could not receive follow-up during the outbreak. They were deeply concerned about several issues common to preterm infants at home, including how to feed, oral medicine and nutritional supplement adjustment, growth and development. Though COVID-19 has seriously affected everyone's lives, follow-up with preterm infants is still very important and necessary. Little is known about the optimal management of follow-up for preterm infants during the epidemic.

The development of various forms of internet medical services has played a positive role in reducing the burden of clinics, facilitating patients, and reducing the difficulty of seeing a doctor. Nevertheless, there are few relevant studies reporting the follow-up of premature infants using online medical services after discharge in China. During the epidemic, were the parents of preterm babies anxious about the follow-up with their babies? What were they concerned about? Is online follow-up an alternative way to answer most questions for parents of preterm babies? We sought to answer these questions.

In our study, the same group of doctors who followed up with preterm babies face-to-face before the epidemic conducted online follow-up. The contents of online and face-to-face follow-up visits were basically the same. Most problems, such as growth monitoring and evaluation, feeding consultation and guidance, nutrition supplementation, nursing and disease prevention guidance, and early development promotion guidance, can be addressed via online follow-up. Neurodevelopmental milestones can be observed via WeChat video with the help and cooperation of parents, as mentioned above.

The results of this study showed that the anxiety level of the preterm infants' families was significantly reduced through online follow-up, most parents were satisfied or very satisfied with online follow-up, and most thought that online follow-up had answered all their questions. However, online follow-up was mainly through parents' reports and video observation. Although there was guidance from doctors, parents' skills were limited. Some items, such as the evaluation of muscle tension and hip joints, might not be accurate. For those babies who were suspected to have problems, we asked their parents to see doctors face-to-face with their babies.

However, the survey after online follow-up illustrated that only 35.5% of parents thought that online follow-up could replace face-to-face follow-up. Doctors were unable to auscultate, palpate and evaluate physical activities, and blood tests, ultrasound and fundus examination could not be completed online. We conducted a combination of online and face-to-face follow-up to ensure the completion of all follow-up contents and take the safety and convenience of the infants into account. There were 14 premature infants who received face-to-face follow-up within 3 weeks after discharge because of special laboratory tests or examinations and 21 who received follow-up online. After that, parents of preterm infants consulted the doctors on test results and medication guidance via online follow-up, while preterm infants with abnormalities found in online follow-up were referred for face-to-face follow-up. The first-time follow-up rate of preterm infants in our study was as good as that in the same period of the previous year, which demonstrated that most preterm infants received follow-up. Parental anxiety was relieved during the epidemic.

In recent years, there have been only a few reports on the follow-up rate of preterm infants. Studies have shown that the first-time follow-up rate of preterm infants in China may be similar to that in developed countries, but the follow-up compliance afterward should be improved (8, 10). COVID-19 was quickly brought under control in China. Since April, our outpatient department has gradually recovered, and more and more parents of preterm infants have chosen to visit the clinic. Therefore, our online follow-up study was closed by the end of April, and only the first-time follow-up rate after discharge was calculated.

In our study, 2 patients were lost to follow-up before the first follow-up visit. We were unable to determine whether they went to other institutions. To ensure follow-up compliance, Western countries established collaboration networks (8, 11, 12), which was an advanced management pattern from which much can be learned. Using identical standards, the medical data of preterm infants were input from admission, and the follow-up medical records were recorded in the clinics of all medical institutes, which was conducive to ensuring the continuity and homogeneity of follow-up.

Some clinics in western countries reported their efforts in the follow-up of preterm infants during the COVID-19 epidemic (13, 14). Similarly to our online follow-up, they conducted telemedicine or hybrid virtual follow-up model to better adapt to the outbreak. The follow-up attendance rate was increased (13), and families were satisfied with the follow-up via telemedicine (14).

Since August 2020, PUMCH has carried out online medical services through its official app, providing medical services for patients over 6 years old who have visited the hospital within the previous 6 months. In the near future, the combination of online and face-to-face follow-up will also become the main form of follow-up for preterm infants, which can not only address parents' concerns but also ensure that they can visit clinics with their infants for follow-up at key time points.

Our study aimed to explore whether the combination of online and face-to face follow-up during special periods can guarantee the follow-up rate and relieve the anxiety of the family, so we did not compare the health outcomes between online and face-to-face follow-up of preterm infants, which is one of the limitations of our study. Furthermore, online follow-up was offered to parents and accepted by those who felt a need for it; thus, responding parents may not be representative of the whole group. The sudden epidemic required us to take immediate measures. Therefore, the questionnaires might not cover all the problems that parents were concerned about. However, if doctors asked parents about those problems, such as colic, abdominal distention, regurgitation, startle and sleep issues during online follow-up, they would answer and guide the parents thoroughly. The results of questionnaires sent before and after online follow-up showed that the anxiety level of parents significantly declined. In addition, our questionnaires did not collect data on the socioeconomic status and level of education of the participants, which might have caused selection bias.

## Conclusions

The combination of online and face-to-face follow-up solved the problem of follow-up for preterm infants during the outbreak, alleviated the anxiety of the parents, and achieved a similar first-time follow-up rate as the same period of last year. In the future, online and face-to-face follow-up can be combined to address parents' concerns and ensure the quality of follow-up.

## Data Availability Statement

The data analyzed in this study is subject to the following licenses/restrictions: The dataset will be available if requested. Requests to access these datasets should be directed to lilinlin@pumch.cn.

## Ethics Statement

The study is exempt from full Institutional Review Board review because it only involves surveys, standard educations tests, observation of public behavior. The protocol number is SK-1392. Written informed consent to participate in this study was provided by the participants' legal guardian/next of kin.

## Author Contributions

ZL conceived the study, collected data, and edited the manuscript. LL assisted the study design, collected data, carried out statistical analysis interpreted results, and wrote the manuscript. WW assisted the study design, edited the manuscript, and collected data. JL participated in collecting data, interpretation of the results, and edited the manuscript. YZ, CW, and LW assisted the study design participated in collecting data. All authors read and approved the final manuscript.

## Conflict of Interest

The authors declare that the research was conducted in the absence of any commercial or financial relationships that could be construed as a potential conflict of interest.
